# Biochemical metabolism of young plants of Ucuúba (*Virola surinamensis*) in the presence of cadmium

**DOI:** 10.1186/s12870-021-02912-y

**Published:** 2021-03-24

**Authors:** W. V. Andrade Júnior, C. F. Oliveira Neto, B. G. Santos Filho, E. D. Cruz, C. B. Amarante, A. V. C. Barbosa, G. A. S. Nogueira, V. R. Nascimento, D. J. P. Sousa, J. S. S. Teixeira

**Affiliations:** 1 Federal Rural University of the Amazon, Institute of Agronomists Sciences, Campus Belém, Belém, Pará Brazil; 2 Brazilian Agricultural Research Corporation (Embrapa), Belem, Pará Brazil; 3 Museu Paraense Emílio Goeldi (MPEG), Belém, Brazil; 4 Federal Rural University of the Amazon, Institute of Agronomists Sciences, Campus Parauapebas, Parauapebas, Pará Brazil

**Keywords:** Nitrate reductase, Total soluble carbohydrates, Proline, Sucrose, Reducing sugars

## Abstract

*Virola surinamensis* is a forest species widely distributed in the estuaries of the Amazon. These ecosystems are susceptible to contamination by Cadmium (Cd), indicating that the plant has strategies for tolerating this metal. The aim of this study was to assess the nitrogen and carbon metabolism of young plants of Ucuúba (*Virola surinamensis*) in the presence of cadmium with the perspective of the phytoremediation of contaminated environments. The used experimental design was a completely randomized design with five Cd concentrations (0, 15, 30, 45, and 60 mg L^− 1^), for 60 days. In general, Cd did not affect nitrate concentration in the root but had a positive effect on leaves. The reduction of nitrate reductase (NR) in plants exposed to Cd was followed by a decrease in ammonia, total soluble amino acids (TSA), and total soluble proteins (TSP). Cd promoted an increase in the concentration of total soluble carbohydrates (TSC), proline, sucrose, and reducing sugars in the plants. The increase in TSC, sucrose and proline, suggests a metabolic regulatory mechanism of *V. surinamensis* against Cd stress.

## Background

Increased cadmium (Cd) concentration in the environment, caused especially by mining residues and excessive use of phosphate fertilizers, promotes serious imbalances in terrestrial and aquatic ecosystems because it is highly toxic and persistent in the environment, as well as present a high mobility in the soil for plants, being incorporated and bioaccumulated to other components of the food chain, rapidly affecting the growing number of organisms [1].

High levels of Cd in the soil commonly causes many stress symptoms in plants such alterations in the concentration of starch and soluble carbohydrates in plants tissues [2, 3]. The lower nitrate absorption (NO_3_^−^) [4, 5], changes in nitrate reductase (NR) activity [6], proline [5, 7], total soluble proteins (PST) and total soluble amino acids (TSA) [8] in plants under the effect of Cd have also been observed.

It has been postulated that higher plants are more sensitive to Cd stress [9]. However, study conducted by Andrade Júnior et al. [10] demonstrated medium and high tolerance of *Virola surinamensis* to Cd. Variations in Cd tolerance in plants may be associated with changes in nitrogen and or carbon metabolism. Differential Cd tolerance can be attributed to differential accumulation of amino acids such as proline, and sugars, which serve as compatible osmolytes and antioxidants or are involved in other plant defense pathways against stress [9].

*V. surinamensis* (Ucuúba) a forest species with economic and medicinal interest, besides being useful for recomposition of altered areas. Is a species widely distributed and adapted to the lowland and igapó ecosystems in the Amazon [10] These ecosystems are constantly susceptible to heavy metal contamination, as Cd [11–13], indicating that the plant has strategies to tolerate environments contaminated by these metals.

Recent study showed that, in addition to tolerance to Cd, *V. surinamensis* had a greater ability to extract and accumulate metal in the root, restricting its transport to the aerial part [10]. Species with these characteristics are promising for phytostabilization of metals. Thus, we tested the hypothesis that *V. surinamensis* develops biochemical strategies capable of tolerating and accumulate high Cd concentrations. Thus, this study aimed to assess the nitrogen and carbon metabolism of young plants of *V. surinamensis* in the presence of cadmium, in order to contribute to understand the potential of *V. surinamensis* against Cd stress.

## Results

### Effect of cd on the concentrations of nitrate, nitrate reductase, and free ammonium

**Fig. 1:**
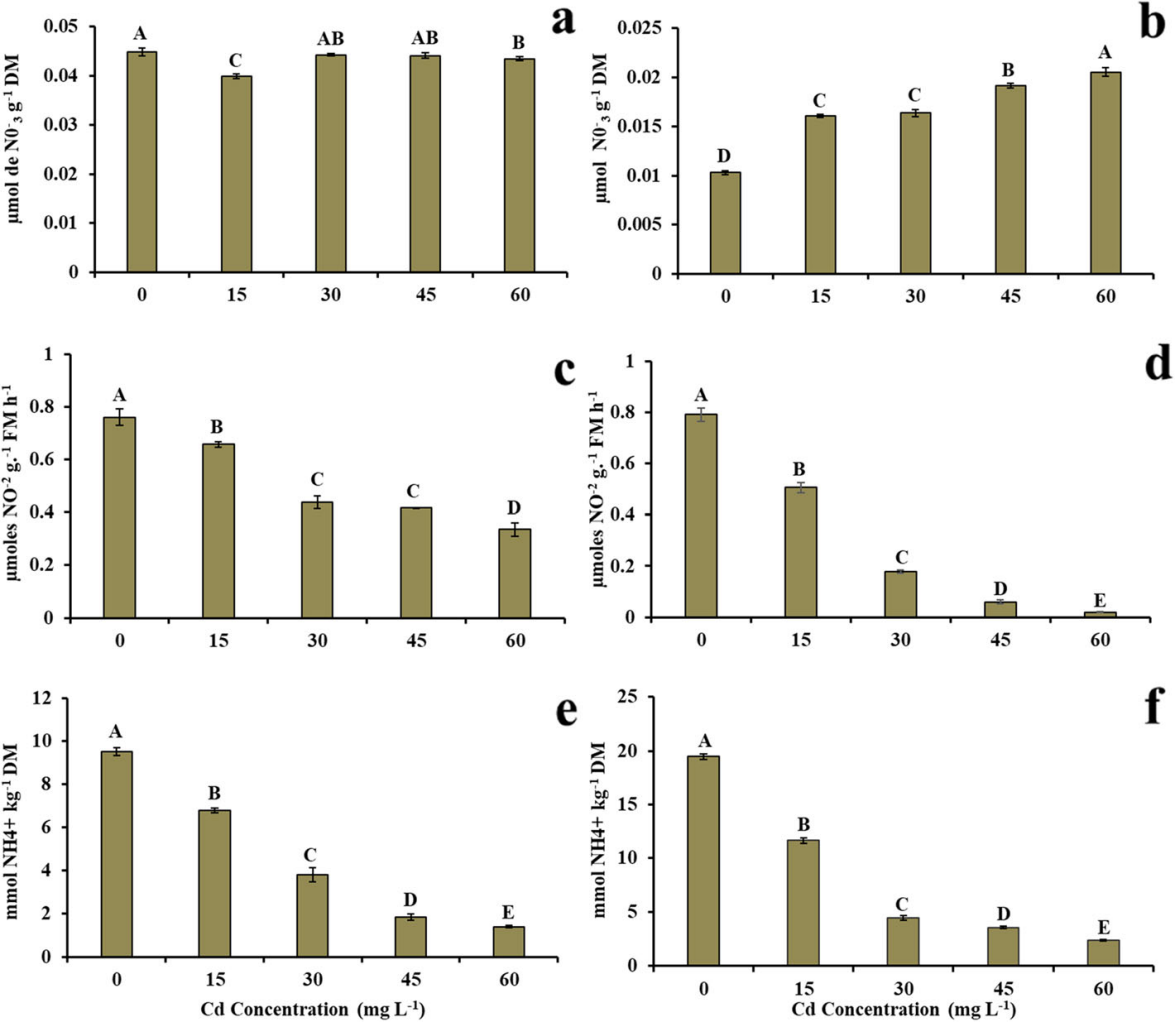
**a** Nitrate concentration in the root, **b** nitrate concentration in the leaves, **c** nitrate reductase concentration in the root, **d** nitrate reductase concentration in the leaves, **e** ammonium concentration in the root, **f** ammonium concentration in the leaves in young plants of *V. surinamensis* exposed to five cadmium concentrations (0, 15, 30, 45, and 60 mg). Different letters for cadmium concentrations in solution indicate significant differences in the Tukey’s test (*P* < 0.05). Mean ± SD, *n* = 7

Nitrate concentrations in the roots were not significantly affected by Cd, except for the dose of 15 mg L^− 1^ of Cd (Fig. 1a). In the leaves, nitrate concentrations were significantly affected by Cd (Fig. 1b). In the roots, Cd concentrations reached 0.045 and 0.04 μmol NO_3_^−^ g^− 1^ DM in the control treatment (0 mg L^− 1^ of Cd) and at a dose of 15 mg L^− 1^ of Cd, respectively (Fig. 1a), corresponding to a reduction of 11.11% when compared to the control. In the leaves, values of 0.01 and 0.02 μmol NO_3_^−^ g^− 1^ DM were obtained in the control plants (0 mg L^− 1^ of Cd) and at the highest dose of Cd (60 mg L^− 1^ of Cd), respectively (Fig. 1b), characterizing an increase of 100% in the treatment of 60 mg L^− 1^ of Cd when compared to the control treatment.

The nitrate reductase activity (NRA) was significantly affected by Cd, both in roots and leaves (Fig. 1c, d). In the roots, the lowest value (0.33 μmol NO_2_^−^ g^− 1^ FM h^− 1^) was observed at a dose of 60 mg L^− 1^ of Cd, representing a 56% reduction when compared to the control treatment (0.76 μmol NO_2_^−^ g^− 1^ FM h^− 1^) (Fig. 1c). The reduction was more accentuated in the leaves, reaching a value of 0.02 μmol NO_2_^−^ g^− 1^ FM h^− 1^ at a dose of 60 mg L^− 1^ of Cd, corresponding a decrease of 97.47% when compared to the control treatment (0.79 μmol NO_2_^−^ g^− 1^ FM h^− 1^) (Fig. 1d).

Cd significantly affected free ammonia, both in roots and leaves (Fig. 1e, f). In the roots, values of 9.52 mmol NH_4_^+^ kg^− 1^ DM (0 mg L^− 1^ of Cd) and 1.39 mmol of NH_4_^+^ kg^− 1^ DM (60 mg L^− 1^ of Cd) were obtained, representing a 85.4% reduction at the highest dose of Cd when compared to the control treatment (Fig. 1e). In the leaves, Cd effect was more significant, promoting a reduction of 87.77% in ammonia concentration at a dose of 60 mg L^− 1^ of Cd (2.38 mmol of NH_4_^+^ kg^− 1^ DM) when compared to the control treatment (19.47 mmol of NH_4_^+^ kg^− 1^ DM) (Fig. 1f).

### Effect of cd on the concentrations of total soluble amino acids, total soluble proteins and proline concentration

**Fig. 2:**
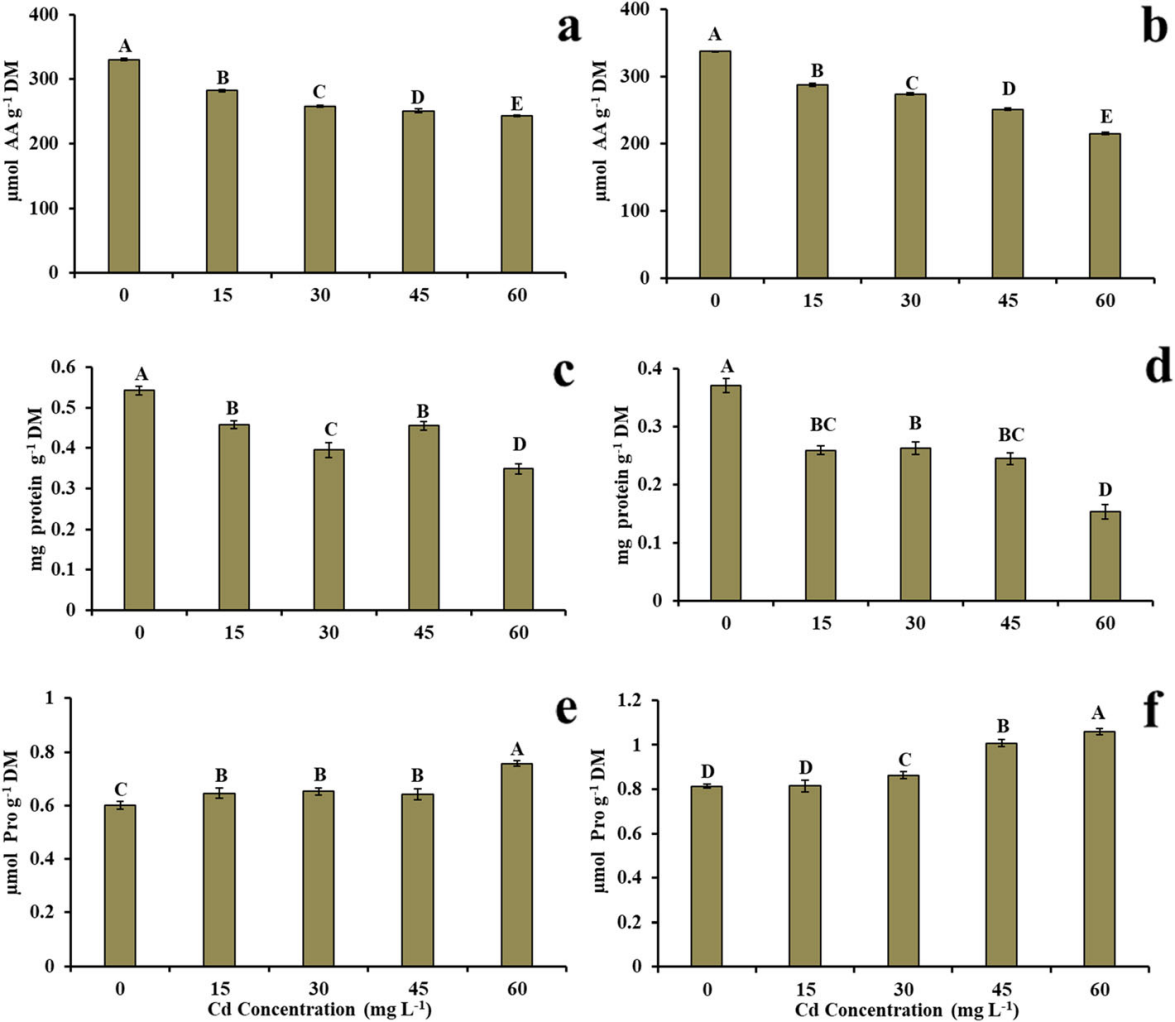
**a** Concentration of total soluble amino acids in the root, **b** concentration of total soluble amino acids in the leaves, **c** concentration of total soluble proteins in the root, **d** concentration of total soluble proteins in the leaves, **e** Proline concentration in the root, **f** proline concentration in the leaves in young plants of *V. surinamensis* exposed to five cadmium concentrations (0, 15, 30, 45, and 60 mg). Different letters for cadmium concentrations in solution indicate significant differences in the Tukey’s test (*P* < 0.05). Mean ± SD, *n* = 7

The concentration of total soluble amino acids in roots and leaves was significantly affected by Cd (Fig. 2). In the roots, the concentration was 330 and 243 μmol AA g^− 1^ DM in the control treatment (0 mg L^− 1^ of Cd) and at a dose of 60 mg L^− 1^ of Cd, respectively (Fig. 2a), corresponding to a reduction of 26.36% at the highest Cd dose when compared to the control treatment. In the leaves, values of 337 and 215 μmol AA g^− 1^ DM were obtained in the control plants (0 mg L^− 1^ of Cd) and at the highest Cd dose (60 mg L^− 1^ of Cd), respectively (Fig. 2b), characterizing a 36.2% reduction in the treatment of 60 mg L^− 1^ of Cd when compared to the control treatment.

The concentrations of total soluble proteins in plants submitted to the presence of Cd were significantly reduced in both roots and leaves (Fig. 2c, d). The highest and lowest concentrations of proteins in roots occurred in the control treatment (0.54 mg protein g^− 1^ DM) and at a dose of 60 mg L^− 1^ of Cd (0.35 mg protein g^− 1^ DM), with a 35.18% reduction at the highest Cd dose when compared to the control treatment (Fig. 2c). In the leaves, the values obtained were 0.37 mg protein g^− 1^ DM (control treatment) and 0.15 mg protein g^− 1^ DM (60 mg L^− 1^ of Cd), corresponding to a decrease of 59.46% in the lowest Cd dose when compared to the control treatment (Fig. 2d).

Proline concentrations in roots and leaves of plants submitted to Cd doses increased significantly (Fig. 2). Values of 0.60 μmol Pro g^− 1^ DM (0 mg L^− 1^ of Cd) and 0.76 μmol Pro g^− 1^ DM (60 mg L^− 1^ of Cd) were obtained in the roots, representing a 26.7% increase at the highest Cd dose when compared to the control treatment (Fig. 2e). In the leaves of control plants and at a dose of 60 mg L^− 1^ of Cd, proline concentrations were 0.81 and 1.06 μmol Pro g^− 1^ DM, respectively, demonstrating a 30.86% increase of proline in plants with the highest Cd dose when compared to the control treatment (Fig. 2f).

### Concentration of total soluble carbohydrates, sucrose, and reducing sugars in the presence of cd

**Fig. 3:**
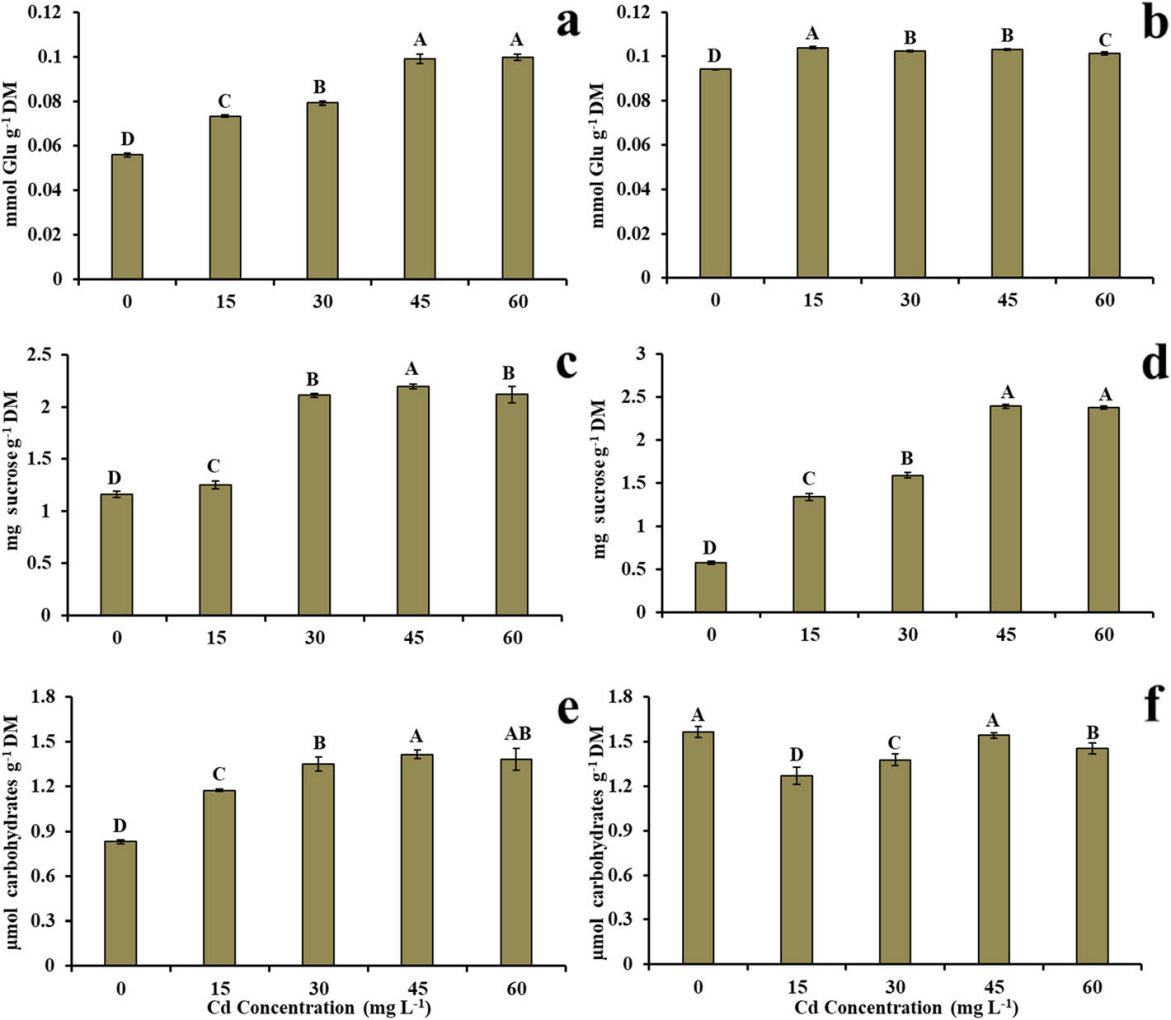
**a** Concentration of total soluble carbohydrates in the root, **b** concentration of total soluble carbohydrates in the leaves, **c** sucrose concentration in the root, **d** sucrose concentration in the leaves, **e** concentration of reducing sugars in the root, **f** concentration of reducing sugars in the leaves in young plants of *V. surinamensis* exposed to five cadmium concentrations (0, 15, 30, 45, and 60 mg). Different letters for cadmium concentrations in solution indicate significant differences in the Tukey’s test (*P* < 0.05). Mean ± SD, *n* = 7

The concentrations of total soluble carbohydrates in Cd-treated plants increased significantly in both roots and leaves (Fig. 3a, b). The lowest and highest concentrations of carbohydrates in the roots were observed in the control treatment (0.06 mmol Glu g^− 1^) and at a dose of 60 mg L^− 1^ of Cd (0.1 mmol Glu g^− 1^), with an increase of 83.3% at the highest Cd dose when compared to the control treatment (Fig. 3a). In the leaves, the obtained values were 0.09 mmol Glu g^− 1^ (control treatment) and 0.1 mmol Glu g^− 1^ (15 mg L^− 1^ of Cd), corresponding to an 11.11% increase at the lowest Cd dose when compared to the control treatment (Fig. 3b).

Sucrose concentrations in Cd-treated plants increased significantly in both roots and leaves (Fig. 3c, d). In the roots, the values were 1.16 mg sucrose g^− 1^ DM (0 mg L^− 1^ of Cd) and 2.11 mg sucrose g^− 1^ DM (60 mg L^− 1^ of Cd), representing an increase of 81.9% at the highest Cd dose when compared to the control treatment (Fig. 3c). The lowest and highest concentrations of sucrose in the leaves were observed in the control treatment (0.57 mg sucrose g^− 1^ DM) and at a dose of 60 mg L^− 1^ of Cd (2.38 mg sucrose g^− 1^ DM), with a 317.54% increase at the highest Cd dose when compared to the control treatment (Fig. 3d).

The concentrations of reducing sugars increased in the roots and reduced significantly in the leaves of plants submitted to the presence of Cd (Fig. 3e, f). Values of 0.83 μmol carb g^− 1^ DM (0 mg L^− 1^ of Cd) and 1.42 μmol carb g^− 1^ DM (45 mg L^− 1^ of Cd) were obtained in the roots, representing a 71.08% increase at a dose of 45 mg L^− 1^ of Cd when compared to the control treatment (Fig. 3e). The concentrations in the leaves of control plants and at a dose of 15 mg L^− 1^ of Cd were 1.57 and 1.27 μmol carb g^− 1^ DM, respectively, demonstrating a 19.11% reduction of reducing sugars in plants with the lowest Cd dose when compared to the control treatment (Fig. 3f).

## Discussion

NO_3_^−^, an important N source, is actively absorbed by the plasma membrane of epidermal and cortical cells of roots through nitrate carrier proteins, but in plants exposed to Cd, there is an inhibition of the activities of these proteins [14] because Cd damages the normal function of the proton pump (H^+^ ATPase) in the plasmalemma [15, 16]. However, in general, no reduction of NO_3_^−^ was observed in the roots of *V. surinamensis* (Fig. 1a), indicating that the presence of Cd probably did not affect the activity of NO_3_^−^ carrier proteins, which is in accordance with the study performed by [17], who showed an increase of the total ATPase in the root and stem of *Cucumis sativus* in the presence of Cd.

In healthy plants, once absorbed by roots, NO_3_^−^ is transported to the leaves, stored in the vacuoles or reduced into nitrite (NO_2_^−^) by NAD(P)H-dependent cytosolic NR activity [18]. In this study, the increase of NO_3_^−^ in the leaves of *V. surinamensis* (Fig. 1b) suggests that Cd did not interfere with the translocation of the nitrogen compound to the shoot. The assimilation of NO_3_^−^ into the cytosol of mesophyll cells may have been affected by the NRA inactivation caused by Cd. The reduction of NRA with the increasing Cd doses in the nutrient solution may be an efficient energy-saving mechanism to reduce the effect of stress and not to decrease NO_3_^−^ in the plant.

NR is the key enzyme in the process of NO_3_^−^ assimilation [5] and is regulated by the presence of NO_3_^−^ [19], its degradation, activation or inactivation. Plants exposed to Cd have a reduced NRA, leading to a decreased NO_3_^−^ assimilation because the metal causes a lower NO_3_^−^ absorption by plant roots [4, 5]. In this study, the marked reduction of NRA with the increasing Cd concentration (Fig. 1c) did not appear to have been caused by substrate availability (NO_3_^−^) since there was no reduction of the nitrogen compound in the plant root and shoot, suggesting a direct effect of Cd on NR activity, i.e. the interaction of the metal with the thiol group (−SH) in the active site of the enzyme would result in the inactivation. Reduction of nitrate reductase activity was also observed in other tree species [5] exposed to Cd.

Ammonium ion is a central intermediate in the metabolism of nitrogen in plant, produced during nitrate assimilation, deamination of amino acids and photorespiration [20]. Considering that from ammonia, there are several biosynthesis routes for all amino acids [21], it can be inferred that in this study, the decrease in ammonia levels (Fig. 1e, f), in plants under Cd, it may be related to the reduction of TSA (Fig. 2a, b) or to the increase in the synthesis of specific amino acids, of protection and stress regulation, such as proline (Fig. 2e, f).

Cd stress in plants causes protein degradation and affects amino acid metabolism [22]. The reduction in TSP (Fig. 2c, d) in *V. surinamensis* under Cd may be due to the activation of proteases that degrade proteins for specific amino acid biosynthesis such as proline (Fig. 2e, f). Thus, the degradation of TSP could function as an important mechanism of self-protection and / or cell signaling against Cd stress. Another explanation for the reduction of TSP in plants exposed to Cd would be the direct effect of the metal on the NRA that affected concentration of TSP. In fact, a significant positive correlation coefficient (*r* = 0.784; *p* = 0.0367) was observed between these variables in plants under Cd, ie the decrease in TSP in *V. surinamensis* under Cd would be associated with a reduction in NRA. The results obtained in present study in relation the total soluble proteins were evidenced by Anand et al. [23].

The highest proline content in plants exposed to Cd occurred by de novo synthesis or decreased degradation and/or both processes [24]. In this study, the increase of proline (Fig. 2a, b) in plants in the presence of Cd may be related to NO_3_^−^ concentration (Fig. 1a, b), since there was a significant positive correlation coefficient (*r* = 0.801; *P* = 0.0304) between these variables, indicating that the increase in proline in plants with Cd is associated to the increase in NO_3_^−^. On the other hand, it has been reported that to degradation of proteins by proteolytic enzymes [25] and the accumulation of this amino acid and formation of a non-toxic Cd-proline complex in tissues would be a plant response to reduce the phytotoxicity of the metal [26, 27]. The increase of proline induced by Cd was evidenced in other forest species [3, 7, 28].

The increase of TSC in *V. surinamensis* exposed to Cd (Fig. 3a, b) may have worked as a compatible solute, which would help the plant in the osmotic adjustment against Cd stress [24], i.e. the accumulation of TSC may have contributed to the maintenance of the water status of the plant, favoring tissue protection and physiological processes, which is an important mechanism in the tolerance of *V. surinamensis* to the presence of Cd, at least during the experimental period. The results obtained in present study in relation to total soluble carbohydrates were evidenced by Anand et al. [23].

Sucrose is a disaccharide consisting of glucose and fructose and, by means of the invertase activity, plays an important metabolic role as a donor of glycosyl and fructosyl for the synthesis of polysaccharides [29] and amino acids in plants [30]. Therefore, the increase in sucrose concentration (Fig. 3c, d) in *V. surinamensis* exposed to Cd may be due to the inhibition of invertase activity, interfering with carbon and nitrogen metabolism, especially in proline accumulation (Fig. 2a, b). Another explanation for sucrose accumulation would be because the metal positively affects the activity of sucrose phosphate synthase (SPS) and negatively affects the sucrose synthase (SuSy) [31]. In addition, the increase in sucrose concentration in *V. surinamensis* exposed to Cd may be related to the degradation of starch by the activity of the enzymes α- and β-amylase hydrolases although heavy metals have an inhibitory effect on these enzymes [32]. The higher concentration of sucrose in the plant exposed to Cd could be related to a reduction in the cell metabolism of this carbohydrate [33] as a form of energy saving since sucrose accumulation in plants submitted to Cd would be a form of tolerance to the metal [8], which is attributed to chelation of Cd by sucrose. Thus, high concentrations of sucrose in *V. surinamensis* suggest a good metabolic regulatory state of the plant in the presence of Cd. The high concentration of sucrose was also observed in other species of plants exposed to Cd [34, 35].

The highest concentration of reducing sugars in plants under stress caused by Cd (Fig. 3e, f) indicates energy savings by plants or even the presence of Cd negatively affecting cell respiration of root and shoot. The results are consistent with those obtained by Xie et al. [9], who suggested the increase of reducing sugars due to the lower utilization of these carbohydrates in plants exposed to Cd. The highest accumulation of reducing sugar in the root (Fig. 3e) suggests an increase in the transport of these carbohydrates from the shoot to the growing cells of the root system, indicating that Cd may not have affected the transport system of assimilates of *V. surinamensis*. In addition, the sugar transported to the roots because of starch degradation would be an essential energy substrate for the resumption of respiration, conferring a mechanism of tolerance of the plant against the phytotoxic effect of Cd [8]. Similar results in the reducing sugar concentration have been found in other species [36].

## Conclusion

The effect of Cd was evidenced by the reduction of NR, ammonia, TSA and TSP activity, suggesting metal toxicity, at least in part, in the assimilation and nitrogen metabolism of *V. surinamensis*.

The increase in TSC, sucrose and proline, suggests a metabolic regulatory mechanism in *V. sirinamensis* in the presence of Cd.

## Methods

### Experiment location

The experiment was conducted in a greenhouse at the Federal Rural University of Amazonia (UFRA) in Belém, State of Pará, Brazil. Belonging to the Institute of Agricultural Sciences with the following geographic coordinates (01°27′21″ S, 48°30′16″ W) and in the established period from 15 September 2017 to 14 November 2017.

### Plant material and growth condition

Seeds of *V. surinamensis* were collected in the area of the Brazilian Agricultural Research Corporation (Embrapa Eastern Amazon), located in Belém, State of Pará, Brazil (01°26′44.2″ S, 48°25′03.8″ W). The identification and collection of seed samples for the experiment was carried out by the team of Dr. Eniel Cruz, a researcher at the Brazilian Agricultural Research Corporation, Agroforestry Research Center of the Eastern Amazon. To collect seeds from this area, authorization is not necessary as it is not a Forest Reserve. At the time of collection, no botanical sample was taken for the IAN Herbarium of Embrapa Amazônia Oriental because it is a very common species and is easily identified.

The identifications were carried out by the employees of Embrapa Amazônia Oriental, in relation to the verification of the samples, the herbarium of this institution is in quarantine due to COVID-19, with no expected return. Each country has its own rules of access to its genetic resources and in Brazil this access is more flexible for Universities and Research Institutions.

These seeds were sown in 5^−^L polyethylene trays containing sand and sterilized sawdust (1:1, v/v), and maintained under mean air temperature (Tair) and relative air humidity (RH) of 28 °C and 90%. After emergence, the seedlings containing the first pair of eophylls were transplanted to 10^−^L polyethylene pots containing yellow latosol and poultry litter (3:1, v/v). The seedlings grown were in a greenhouse for 180 days, being irrigated daily to replace the water lost by evapotranspiration. Subsequently, the young plants were removed and their roots washed with deionized water and transferred to 5^−^L Leonard pots containing sterilized and washed sand and 800 mL of nutrient solution, replaced weekly and constituted of (μM): KH_2_PO_4_, 400; KNO_3_, 2000; Ca (NO_3_)_2_・4H_2_O, 2000; MgSO_4_・7H_2_O, 800; FeEDTA, 400; H_3_BO_3_, 400; MnCl_2_・4H_2_O, 400; ZnCl_2_, 400; CuCl_2_・2H_2_O, 400; and H_2_MoO_4_・H_2_O, 400. The pH was maintained at 5.9 ± 0.2 using HCl and NaOH. The ionic strength was initiated in 25% (10 days) and then increased to 50% (35 days), remaining for a period of acclimatization of 45 days.

### Experimental design and treatment evaluation

After 45 days of cultivation, we selected the most uniform seedling considering height, stem diameter, number of leaves and submitted to five Cd concentrations (treatments) as following: 0 mg L^− 1^ of CdCl_2_ (control), 15, 30, 45, and 60 mg L^− 1^ of CdCl_2_. The doses of Cd were determined based on the Resolution 420 of the National Council of the Environment, which establishes criteria and guiding values of soil quality regarding the presence of chemical substances. The experimental design was a completely randomized design with seven replications, per each treatment, totaling 35 experimental units. As ingle plant per pot was considered a replicate. All variables for treatment comparisons were assessed 60 days after Cd treatment differentiation.

### Biochemical assessments

The biochemical analyses were performed at the Laboratory of EBPS of UFRA. The following variables were determined: contents of nitrate (NO_3_^−^) and free ammonium (NH_4_^+^) [37], activity of the enzyme nitrate reductase (RNO_3_^−^) [38]; total soluble amino acids (TSA) [39], total soluble proteins (TSP) [40]; proline [41], total soluble carbohydrates (TSC) [42]; sucrose [43]; and reducing sugars [44].

### Data analysis

The experimental data were evaluated for normality and homogeneity of variances by the Shapiro-Wilk and Bartlett tests, respectively. The analyzes were performed using the SAS 9.1.3 software [45] and the Rstudio 1.1.383 software. For the parametric variables, the comparison of means was performed in PROC GLM, from SAS, with the method of least squares of adjustment of general linear models, with the MEANS instruction of multiple comparisons for means of main effect with the command *lines tukey* (Tukey’s studentized range test (HSD). For the correlation analysis, the PROC CORR procedure, of the SAS, was used (Pearson lienar and Spearman moment product, for the parametric and nonparametric variables, respectively), the significance of these correlations was performed by the t test. -student The Kruskal-Wallis test with Bonferroni correction was used for nonparametric variables in the control of Type I error, in RStudio. All statistical analyzes were performed at 5% significance level.

## Data Availability

not applicable.

## Abbreviations

NR: Nitrate Reductase
TSA: Total Soluble Aminoacids
TSP: Total Soluble Proteins
TSC: Total Soluble Carbohydrates
Cd: Cadmium
AA: Aminoacids
ROS: Reactive Oxygen Species
SPS: Sucrose Phosphate Synthase
T air: Air
Temp: Temperature
RH: Relative Air Humidity
NO_3_^−^: Nitrate
RNO_3_^−^: Nitrate Reductase
DM: Dry Mass
FM: Fresh Mass
Glu: Glutamine
NH_4_^+^: Ammonia
NRA: Nitrate Reductase Activity
