# Correction to: Biochemical metabolism of young plants of Ucuúba (*Virola surinamensis*) in the presence of cadmium

**DOI:** 10.1186/s12870-021-02950-6

**Published:** 2021-04-08

**Authors:** W. V. Andrade Júnior, C. F. Oliveira Neto, B. G. Santos Filho, E. D. Cruz, C. B. Amarante, A. V. C. Barbosa, G. A. S. Nogueira, V. R. Nascimento, D. J. P. Sousa, J. S. S. Teixeira

**Affiliations:** 1 Federal Rural University of the Amazon, Institute of Agronomists Sciences, Campus Belém, Belém, Pará Brazil; 2 Brazilian Agricultural Research Corporation (Embrapa), Belem, Pará Brazil; 3 Museu Paraense Emílio Goeldi (MPEG), Belém, Brazil; 4 Federal Rural University of the Amazon, Institute of Agronomists Sciences, Campus Parauapebas, Parauapebas, Pará Brazil

**Correction to: BMC Plant Biol 21, 151 (2021)**

**https://doi.org/10.1186/s12870-021-02912-y**

Following publication of the original article [1], the authors identified an error in one of the author names--S. F. Vinícius should have been A. V. C. Barbosa.

The correction does not have any effect on the results or conclusions of the paper. The original article has been corrected.
